# Effect of Vitamin D Supplementation on Bone Mineral Density in Rheumatoid Arthritis Patients With Osteoporosis

**DOI:** 10.3389/fmed.2020.00443

**Published:** 2020-08-21

**Authors:** Oh Chan Kwon, Ji Seon Oh, Min-Chan Park, Yong-Gil Kim

**Affiliations:** ^1^Division of Rheumatology, Department of Internal Medicine, Yonsei University College of Medicine, Seoul, South Korea; ^2^Department of Biomedical Informatics, Asan Medical Center, Seoul, South Korea; ^3^Division of Rheumatology, Department of Medicine, University of Ulsan, College of Medicine, Asan Medical Center, Seoul, South Korea

**Keywords:** rheumatoid arthritis, osteoporosis, vitamin D, supplementation, bone mineral density

## Abstract

**Objectives:** To assess the effect of vitamin D supplementation on bone mineral density (BMD) in rheumatoid arthritis (RA) patients with osteoporosis and determine whether supplementation of more than 800 IU/day, which is the currently recommended dose, is beneficial.

**Methods:** RA patients with osteoporosis who received bisphosphonate were included. Patients were classified into four groups according to the dose of vitamin D supplementation (0, 400, 800, and ≥1,000 IU/day). Multivariable linear regression models were performed to evaluate the effect of each dose of vitamin D supplementation on 1-year% change of BMD.

**Results:** In total, 187 RA patients with osteoporosis were included. In the multivariate model adjusted for potential confounders, patients receiving vitamin D supplementation had a significantly higher increase in 1-year % change in lumbar spine BMD (400 IU/day: β = 2.51 [95% CI: 0.04–4.99], 800 IU/day: β = 2.90 [95% CI: 0.47–5.33], and ≥1,000 IU/day: β = 6.01 [95% CI: 3.71–8.32]) and femoral neck BMD (400 IU/day: β = 3.88 [95% CI: 1.83–5.94], 800 IU/day: β =4.30 [95% CI: 2.25–6.35], and ≥1,000 IU/day: β = 6.79 [95% CI: 4.87–8.71]) than those not receiving the supplementation. Notably, the ≥1,000-IU/day group had a significantly higher increase in 1-year % change in lumbar spine BMD (β = 3.11 [95% CI: 0.86–5.37]) and femoral neck BMD (β = 2.50 [95% CI: 0.63–4.36]) than the 800-IU/day group.

**Conclusion:** In RA patients with osteoporosis receiving bisphosphonates, vitamin D supplementation was associated with a higher increase in BMD. This effect was higher in the vitamin D supplementation dose of ≥1,000 IU/day than in 800 IU/day.

## Introduction

Rheumatoid arthritis (RA) is an autoimmune disease characterized by chronic inflammatory arthritis (1). Since RA is a disease in which long-term glucocorticoid is commonly used (1), glucocorticoid-induced osteoporosis (GIOP) is an important comorbidity that needs to be considered when treating patients with RA. Indeed, population-based studies have reported a higher risk of osteoporosis in patients with RA (2, 3).

In the 2017 American College of Rheumatology guideline for the prevention and treatment of GIOP, a vitamin D intake of 600–800 IU/day has been recommended (4). Similarly, the Korean guideline for the prevention and treatment of GIOP also recommends a vitamin D intake of 800 IU/day (5). However, the recommended vitamin D intake of 600–800 IU/day is based on general population data, rather than those of patients with specific diseases, including RA, who require long-term glucocorticoid therapy (6). Due to the indirectness of the evidence, a vitamin D intake of 600–800 IU/day is only conditionally recommended in the GIOP guideline (4). Currently, data about the effect of vitamin D supplementation on bone mineral density (BMD) in RA patients with osteoporosis are limited, and the optimal dose of supplementation remains unclear.

Osteoporosis is characterized by low BMD and microarchitectural deterioration of the bone tissues, leading to an increased risk of fracture (7). Vitamin D affects the rate of bone turnover and the overall mineralization of the bone. Thus, vitamin D deficiency is associated with a higher bone turnover and incidence of fracture (8). Considering that vitamin D deficiency is highly prevalent in RA patients (9, 10), the required dose of vitamin D supplementation in RA patients with osteoporosis might be higher than the recommended intake of 600–800 IU/day (4), which is based on general population data.

This study aimed to assess the effect of vitamin D supplementation on BMD in RA patients with osteoporosis and to determine whether a dose higher than 800 IU/day is more effective in improving BMD in patients with RA.

## Patients and Methods

### Study Population

Electronic medical records of RA patients who were newly diagnosed with osteoporosis and who started receiving bisphosphonates at two tertiary referral hospitals in Seoul, South Korea, between 2013 and 2017 were retrospectively reviewed. All patients met the 2010 American College of Rheumatology/European League Against Rheumatism classification criteria for RA (11). A diagnosis of osteoporosis was made based on the following BMD results: lumbar spine T-score ≤−2.5 and/or femoral neck T-score ≤−2.5. Patients who were taking medications other than bisphosphonates (selective estrogen receptor modulators, and teriparatide) for the treatment of osteoporosis, those with metabolic diseases, such as thyroid and parathyroid diseases, that can affect BMD, those with a history of previous fracture, and those who were current smokers were excluded.

This study was approved by the Institutional Review Board (IRB) of Gangnam Severance Hospital (IRB No: 3-2020-0043) and Asan Medical Center in Seoul, South Korea (IRB No: 2018-0090). Requirement of informed consent was waived because of the retrospective nature of the study.

### BMD Assessment and Outcome Variables

BMDs of the lumbar spine (first to fourth vertebrae) and femoral neck were evaluated using dual-energy X-ray absorptiometry (GE Healthcare Lunar or Hologic system) (12). As per the insurance policy in our country, BMD was assessed annually (i.e., for insurance coverage, BMD data must be obtained every year). The same instrument was used for repeat BMD measurement in each patient. The BMD results of the Hologic system were converted to GE Healthcare Lunar BMD results using the conversion equation (13). Data about BMD results at the diagnosis of osteoporosis and after 1 year of treatment were collected for analysis. Outcome variables were 1-year % change in lumbar spine and femoral neck BMDs.

### Vitamin D Supplementation

The average dietary intake of vitamin D is relatively low in South Korea (male: 160 IU/day, and female: 104 IU/day) (14), and as the majority of vitamin D intake comes from supplementation rather than diet, we focused on the dose of supplementation of vitamin D. Vitamin D supplements were in the form of calcium–vitamin D complex tablet or bisphosphonate–vitamin D complex tablet. Each tablet contained a specific amount of vitamin D [calcium–vitamin D complex for daily use: 400, 800, and 1,000 IU/tablet; bisphosphonate–vitamin D complex for weekly use: 5,600 IU/tablet [equivalent to 800 IU/day] (15); or bisphosphonate–vitamin D complex for monthly use: 24,000 IU/tablet [equivalent to 800 IU/day] (15)]. Thus, the vitamin D supplementation dose was used as a categorical variable rather than a continuous variable. The type of vitamin D supplement was chosen based on the treating physicians' preference. Patients were classified into four groups according to the dosage of vitamin D supplementation, which were as follows: 0, 400, 800, and ≥1,000 IU/day. As calcium supplementation was provided in the form of calcium–vitamin D complex tablet, the 0-IU/day group also did not receive any calcium supplementation, whereas the other three groups received 500–1,000 mg/day of calcium supplementation in the form of calcium–vitamin D complex tablet. The average dietary intake of calcium in South Korea is 542 mg/day (16). Therefore, the total calcium intake (total of diet and supplementation) of patients receiving vitamin D supplementation was ~1,000–1,500 mg/day, which was in accordance with the recommended calcium intake of 1,000–1,200 mg/day (4).

### Covariates

At the time of diagnosis of osteoporosis, the following data were collected: age, sex, body mass index (BMI), positivity to rheumatoid factor (RF) and anti-cyclic citrullinated peptide (CCP) antibody, and disease activity score 28 with erythrocyte sedimentation rate (DAS28-ESR). Data about medications prescribed during the 1-year interval between the BMD tests, including cumulative dose of glucocorticoid, use of conventional synthetic disease-modifying anti-rheumatic drugs (csDMARDs) and biologic DMARDs (bDMARDs), and type of bisphosphonate, were reviewed. For glucocorticoid use, the total glucocorticoid dose prior to the initial BMD test, and the current status (users or not) of glucocorticoid use at the initial BMD test were also reviewed.

### Statistical Analysis

To compare the different groups, ANOVA was used for continuous variables and the chi-square test (when <20% of cells had expected count <5) or Fisher's exact test (when 20% or more cells had expected count <5) was used for categorical variables. Multivariable linear regression analyses were performed to assess the effect of vitamin D supplementation on 1-year % changes in BMD. For each category of vitamin D supplementation dosage, we estimated the mean difference (effect estimate [β] and 95% confidence interval [CI]) in 1-year % changes in BMD between patients with and without vitamin D supplementation. Potential confounders, such as age, sex, BMI, total glucocorticoid dose prior to the initial BMD test, cumulative glucocorticoid dose between BMD measurements, use of csDMARDs and bDMARDs, DAS28-ESR, and type of bisphosphonate, were adjusted in the multivariable models using enter method. The variation inflation factor (VIF) was assessed to exclude multicollinearity among covariates included in the multivariable analyses. VIFs of all covariates were less than 5, confirming the absence of multicollinearity. The normality of the residual was tested using a histogram and normal P–P plot of the regression standardized residual, and the homoscedasticity of the residual was assessed using the scatter plot: the residual followed a normal distribution and was homoscedastic. Further, to evaluate whether a vitamin D supplementation dose of >800 IU/day is more effective than that of 800 IU/day in terms of improving BMD, the mean difference in 1-year % change in BMD between the ≥1,000- and 800-IU/day groups was assessed. *P* < 0.05 was considered statistically significant. All analyses were conducted using the SPSS software (version 25.0; IBM Corporation, Armonk, NY, USA).

## Results

### Comparison Among the Different Vitamin D Supplementation Dose Groups

A total of 286 patients with RA, who were newly diagnosed with osteoporosis, were identified. Thirty-two patients who received medication other than bisphosphonate (selective estrogen receptor modulators: 30 patients, and teriparatide: 2 patients) for the treatment of osteoporosis, 14 patients with thyroid disease, 2 patients with parathyroid disease, 12 patients with a history of previous fracture, and 39 patients who were current smoker were excluded. The remaining 187 RA patients with osteoporosis were included in the analysis. Sixty-one patients did not receive vitamin D supplementation (0-IU/day group), whereas 23, 73, and 30 patients received vitamin D supplementation at a dose of 400 IU/day (400-IU/day group), 800 IU/day (800-IU/day group), and ≥1,000 IU/day (≥1,000-IU/day group), respectively. In the ≥1,000-IU/day group, 26 (86.7%) patients received 1,000 IU/day, one (3.3%) patient received 1,200 IU/day, and three (10.0%) patients received 1,800 IU/day. Comparison results among the different groups are shown in Table 1. The four groups did not differ in terms of age (*p* = 0.641), sex distribution (*p* = 0.570), BMI (*p* = 0.915), positivity to RF (*p* = 0.633) and anti-CCP antibody (*p* = 0.248), total glucocorticoid dose prior to the initial BMD test (*p* = 0.605), proportion of current glucocorticoid users (*p* = 0.109), cumulative dose of glucocorticoid between BMD tests (*p* = 0.246), use of csDMARDs (methotrexate, *p* = 0.416; hydroxychloroquine, *p* = 0.752; sulfasalazine, *p* = 0.731; leflunomide, *p* = 0.621; and tacrolimus, *p* = 0.946) and bDMARDs (tumor necrosis factor inhibitor, *p* = 0.134; tocilizumab, *p* = 0.610; and abatacept, *p* = 0.851), and DAS28-ESR (*p* = 0.846). The type of bisphosphonate used (risedronate: *p* < 0.001, alendronate: *p* < 0.001, and ibandronate: *p* = 0.001) was different among the groups (Table 1).

**Table 1:** Characteristics of patients (*n* = 187) according to the dose of vitamin D supplementation.

	**0 IU/day (*n* = 61)**	**400 IU/day (*n* = 23)**	**800 IU/day (*n* = 73)**	**≥1,000 IU/day (*n* = 30)**	***P***
Age (years), mean ± SD	65.7 ± 8.9	65.7 ± 9.3	64.0 ± 8.5	64.1 ± 8.7	0.641
Female, n (%)	58 (95.1)	22 (95.7)	65 (89.0)	27 (90.0)	0.570^F^
BMI (kg/m^2^), mean ± SD	22.7 ± 3.2	23.0 ± 3.9	23.1 ± 3.1	22.9 ± 3.5	0.915
RF positive, *n* (%)	45 (73.8)	18 (78.3)	60 (82.2)	22 (73.3)	0.633^X^
Anti-CCP Ab positive, *n* (%)	45 (73.8)	19 (82.6)	62 (84.9)	21 (70.0)	0.248^X^
Total glucocorticoid dose prior to the initial	1612.5 (210.0–2033.8)	1435.0 (330.0–2570.0)	1035.0 (275.0–1922.5)	1762.5 (343.1–3490.0)	0.605
BMD test (mg), median (IQR)					
Current glucocorticoid users, *n* (%)	57 (93.4)	21 (91.3)	58 (79.5)	27 (90.0)	0.109
Glucocorticoid^a^ (mg), mean ± SD	1295.2 ± 731.3	1218.1 ± 651.4	1022.2 ± 841.8	1172.3 ± 842.3	0.246
**USE OF CSDMARDS, *N* (%)**					
MTX	52 (85.2)	19 (82.6)	67 (91.8)	25 (83.3)	0.416^F^
HCQ	28 (45.9)	8 (34.8)	30 (41.1)	11 (36.7)	0.752^X^
SSZ	12 (19.7)	7 (30.4)	15 (20.5)	6 (20.0)	0.731^X^
LEF	9 (14.8)	5 (21.7)	9 (12.3)	3 (10.0)	0.621^F^
TAC	4 (6.6)	2 (8.7)	4 (5.5)	2 (6.7)	0.946^F^
**USE OF BDMARDS, *N* (%)**					
TNFi	1 (1.6)	0 (0.0)	6 (8.2)	3 (10.0)	0.134^F^
Tocilizumab	1 (1.6)	0 (0.0)	0 (0.0)	0 (0.0)	0.610^F^
Abatacept	2 (3.3)	0 (0.0)	1 (1.4)	0 (0.0)	0.851^F^
DAS28-ESR, mean ± SD	3.19 ± 1.04	3.11 ± 0.71	3.19 ± 0.93	3.03 ± 0.73	0.846
**BISPHOSPHONATE, *N* (%)**					
Risedronate	38 (62.3)	11 (47.8)	2 (2.7)	9 (30.0)	<0.001^X^
Alendronate	2 (3.3)	10 (43.5)	65 (89.0)	13 (43.3)	<0.001^X^
Ibandronate	21 (34.4)	2 (8.7)	6 (8.2)	8 (26.7)	0.001^X^
**BASELINE BMD, MEAN ± SD**					
Lumbar spine T-score	−2.93 ± 0.84	−2.72 ± 1.23	−2.85 ± 0.96	−2.99 ± 0.95	0.737
Femoral neck T-score	−2.41 ± 0.72	−2.35 ± 0.85	−2.17 ± 0.79	−2.43 ± 0.71	0.252
**BMD AT 1 YEAR, MEAN ± SD**					
Lumbar spine T-score	−2.85 ± 0.86	−2.46 ± 1.26	−2.52 ± 0.98	−2.50 ± 0.99	0.161
Femoral neck T-score	−2.44 ± 0.74	−2.28 ± 0.80	−2.11 ± 0.77	−2.29 ± 0.67	0.093
**1-YEAR % CHANGE, MEAN ± SD**					
Lumbar spine BMD	0.71 ± 5.29	3.89 ± 4.13	5.17 ± 4.93	7.42 ± 3.64	<0.001
Femoral neck BMD	−2.09 ± 4.74	1.82 ± 3.64	2.21 ± 3.00	4.78 ± 4.58	<0.001

a *Cumulative dose (mg of prednisolone or its equivalent) during the 1-year interval between BMD tests*.

F *P value calculated with Fisher's exact test*.

X *P value calculated with chi-square test*.

*BMI, body mass index; RF, rheumatoid factor; anti-CCP Ab, anti-cyclic citrullinated peptide antibody; csDMARDs, conventional synthetic disease-modifying anti-rheumatic drugs; MTX, methotrexate; HCQ, hydroxychloroquine; SSZ, sulfasalazine; LEF, leflunomide; TAC, tacrolimus; bDMARDs, biologic disease-modifying anti-rheumatic drugs; TNFi, tumor necrosis factor inhibitor; DAS28-ESR, disease activity score 28-erythrocyte sedimentation rate; BMD, bone mineral density*.

**Figure 1:**
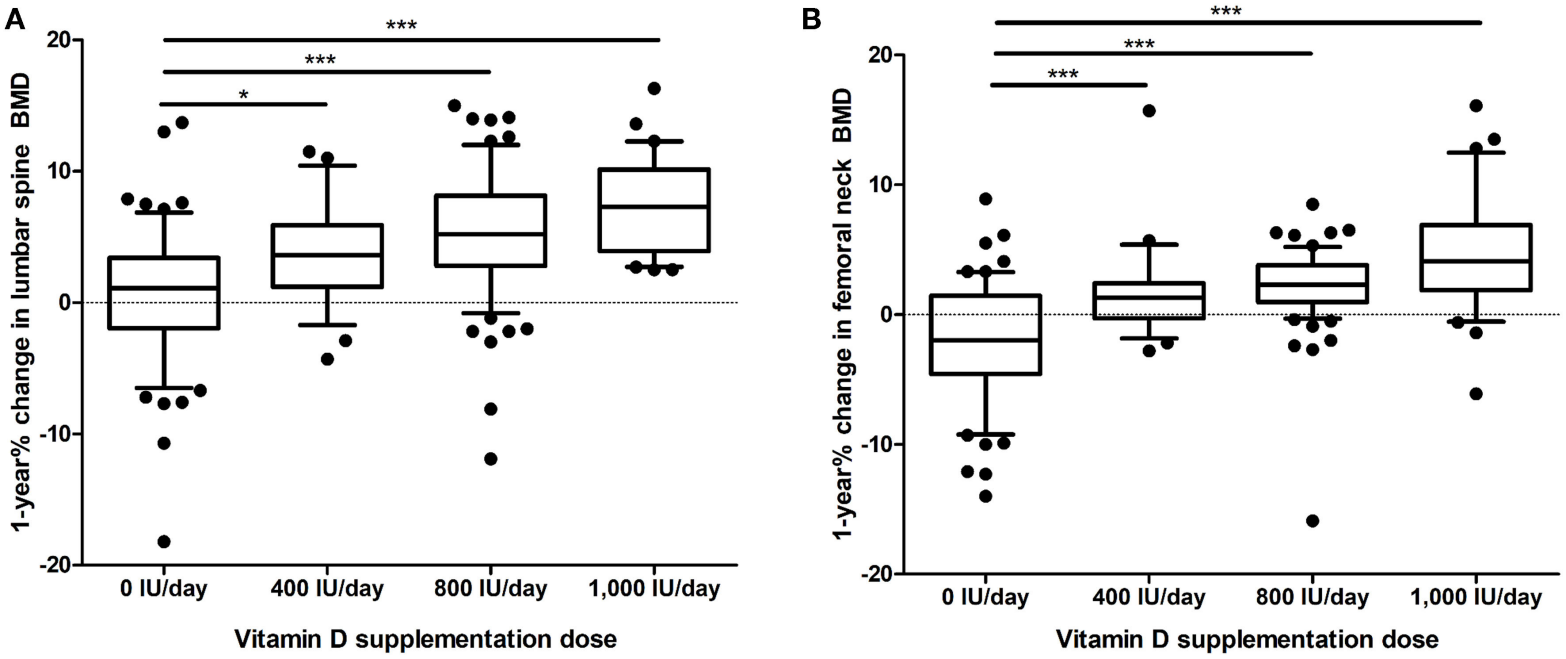
One year % change in **(A)**, lumbar spine BMD and **(B)**, femoral neck BMD according to the dose of vitamin D supplementation. The whiskers represent the 10–90 percentile range, and the black dots represent values outside the 10–90 percentile range. *p < 0.05, ***p < 0.001. BMD, bone mineral density.

According to the initial BMD, 94 (50.3%) patients had osteoporosis in the lumbar spine, 33 (17.6%) patients had osteoporosis in the femoral neck, and 60 (32.1%) patients had osteoporosis in both lumbar spine and femoral neck. The initial lumbar spine T-score (*p* = 0.737) and initial femoral neck T-score (*p* = 0.252) did not differ among the four groups. The mean values of 1-year % changes in the lumbar spine BMD and femoral neck BMD in the total study population were 3.92 and 1.19%, respectively. The 1-year % change of lumbar spine BMD (*p* < 0.001) and femoral neck BMD (*p* < 0.001) was significantly different among groups (Table 1 and Figure 1).

### Effect of Vitamin D Supplementation on 1-year % Changes in BMD

The results of linear regression analyses are shown in Table 2. The overall p value for the regression was *p* < 0.001 for all univariable and multivariable analyses. The total amount of variance explained in the univariable and in the multivariable analyses was as follows: univariable (lumbar spine), 0.210; multivariable (lumbar spine), 0.298; univariable (femoral neck), 0.281; and multivariable (femoral neck), 0.370. In the multivariable analysis, the 1-year % change in lumbar spine BMD was significantly higher in the vitamin D supplementation groups (400 IU/day: β = 2.51 [95% CI 0.04–4.99], *p* = 0.047; 800 IU/day: β = 2.90 [95% CI 0.47–5.33], *p* = 0.020; and ≥1,000 IU/day: β = 6.01 [95% CI 3.71–8.32], *p* < 0.001) than in the 0-IU/day group. Similarly, the supplementation of vitamin D, regardless of dose, was significantly associated with a higher 1-year % change in femoral neck BMD (400 IU/day: β = 3.88 [95% CI 1.83–5.94], *p* < 0.001; 800 IU/day: β = 4.30 [95% CI 2.25–6.35], *p* < 0.001; and ≥1,000 IU/day: β = 6.79 [95% CI 4.87–8.71], *p* < 0.001) (Table 2). Coefficients and their significance levels of all variables included in the multivariable analysis are shown in Supplementary Table 1.

**Table 2:** Linear regression model estimates of the effect of vitamin D supplementation based on 1-year % changes in BMD.

	**Univariable analysis**		**Multivariable analysis^a^**	
	**β (95% CI)**	***P***	**β (95% CI)**	***P***
**LUMBAR SPINE**				
0 IU/day	0 (ref.)		0 (ref.)	
400 IU/day	3.18 (0.87–5.49)	0.007	2.51 (0.04–4.99)	0.047
800 IU/day	4.46 (2.82–6.10)	<0.001	2.90 (0.47–5.33)	0.020
≥1,000 IU/day	6.71 (4.61–8.82)	<0.001	6.01 (3.71–8.32)	<0.001
**FEMORAL NECK**				
0 IU/day	0 (ref.)		0 (ref.)	
400 IU/day	3.92 (1.99–5.84)	<0.001	3.88 (1.83–5.94)	<0.001
800 IU/day	4.30 (2.94–5.67)	<0.001	4.30 (2.25–6.35)	<0.001
≥1,000 IU/day	6.88 (5.12–8.63)	<0.001	6.79 (4.87–8.71)	<0.001

a *Multivariable model adjusted for age, sex, BMI, total glucocorticoid dose prior to the initial BMD test, cumulative glucocorticoid dose between BMD tests, use of csDMARDs and bDMARDs, DAS28-ESR, and type of bisphosphonate*.

### Comparison of 1-year % Changes in BMD Between the ≥1,000- and 800-IU/day Groups

In the multivariable linear regression analysis comparing the effects of ≥1,000 and 800 IU/day, a vitamin D supplementation of ≥1,000 IU/day was associated with a significantly higher 1-year % change in lumbar spine BMD (β = 3.11 [95% CI 0.86–5.37], *p* = 0.007) and femoral neck BMD (β = 2.50 [95% CI 0.63–4.36], *p* = 0.009) (Table 3).

**Table 3:** Linear regression model estimates of the difference in 1-year % changes in BMD between the ≥1,000- and 800-IU/day groups.

	**Univariable analysis**		**Multivariable analysis^a^**	
	**β (95% CI)**	***P***	**β (95% CI)**	***P***
**LUMBAR SPINE**				
800 IU/day	0 (ref.)		0 (ref.)	
≥1,000 IU/day	2.25 (0.21–4.30)	0.031	3.11 (0.86–5.37)	0.007
**FEMORAL NECK**				
800 IU/day	0 (ref.)		0 (ref.)	
≥1,000 IU/day	2.57 (0.87–4.27)	0.003	2.50 (0.63–4.36)	0.009

a *Multivariable model adjusted for age, sex, BMI, total glucocorticoid dose prior to the initial BMD test, cumulative glucocorticoid dose between BMD tests, use of csDMARDs and bDMARDs, DAS28-ESR, and type of bisphosphonate*.

## Discussion

In this retrospective study, we showed that in RA patients with osteoporosis who were receiving bisphosphonates, the supplementation of vitamin D was significantly associated with a higher increase in lumbar spine and femoral neck BMDs within 1 year. The mean differences in 1-year % changes in lumbar spine and femoral neck BMD were the highest in the ≥1,000-IU/day group. Notably, ≥1,000 IU/day of vitamin D supplementation was associated with a 3.11% (95% CI: 0.86–5.37%) and 2.50% (95% CI: 0.63–4.36%) higher increase in 1-year % changes in lumbar spine and femoral neck BMD, respectively, compared with 800 IU/day of vitamin D supplementation. This finding has an important clinical implication in that it indicates that a vitamin D supplementation dose higher than that recommended in the current GIOP guideline (4) might be beneficial in terms of improving BMD in RA patients with osteoporosis.

In previous studies evaluating the effect of vitamin D supplementation in non-osteoporotic participants, a 1-year % change in BMD was 1.0–2.5% higher in patients receiving vitamin D supplementation (400–800 IU/day) than in those receiving placebo (17–19). The observed mean difference in 1-year % change in BMD between the ≥1000-IU/day group and the 0-IU/day group in our study was remarkably higher (6.01 and 6.79% in the lumbar spine and femoral neck BMD, respectively). Even in the comparison between the 400-IU/day group and the 0-IU/day group, the mean difference in 1-year % change in BMD was 2.51% and 3.88% in the lumbar spine and femoral neck BMD, respectively. The relatively higher mean difference in 1-year % change in BMD by vitamin D supplementation observed in our data is likely because we only included patients with osteoporosis, whereas the previous studies have included participants who did not present with osteoporosis and the effect of vitamin D supplementation was assessed for preventive measure in these studies (17–19). Moreover, patients not receiving vitamin D supplementation also did not receive calcium supplementation, which may also explain the higher magnitude of 1-year % change in BMD in our study.

In vitamin D deficiency, 1, 25-(OH)-vitamin D interacts with receptors in the osteoblasts, thereby leading to the increased formation of osteoclasts (20). The mature osteoclast then releases enzymes to break down the bone matrix, ultimately releasing calcium and other minerals into the circulation (21). Therefore, vitamin D supplementation is important in the treatment of osteoporosis (22). The higher increase in BMD observed in patients receiving a vitamin D supplementation dose higher than the dose (600–800 IU/day) recommended in the general osteoporosis patients might be attributable to the fact that patients with RA have a higher prevalence and more severe degree of vitamin D deficiency than the general population (9, 10, 23).

A previous study has shown that in trials in which a mean 25-(OH)-vitamin D level of 100 nmol/L was achieved, optimal prevention of osteoporotic fracture was observed (22). In studies in which baseline 25-(OH)-vitamin D levels were between 40 and 77 nmol/L, patients achieved a 25-(OH)-vitamin D level of 100 nmol/L with the supplementation of vitamin D at a dose of 700–800 IU/day (18, 24), whereas in studies in which the baseline levels were as low as 21–26 nmol/L, patients failed to achieve a 25-(OH)-vitamin D level of 100 nmol/L with the supplementation of vitamin D at a dose of 800 IU/day (19, 25). These findings support the notion that patients with vitamin D deficiency, such as those with RA, may benefit from a vitamin D supplementation dose higher than 800 IU/day to improve BMD. Moreover, considering that vitamin D deficiency is inversely associated with the disease activity of RA (9, 10), vitamin D supplementation at a higher dose might be beneficial in improving not only BMD but also the disease activity of RA. However, as vitamin D intoxication can result in the mobilization of skeletal calcium, leading to bone demineralization (26), increasing the dose of vitamin D supplementation indefinitely cannot be advocated. The suggested upper limit of vitamin D intake is 4,000 IU/day (6).

Treatment of RA may affect BMD in RA patients with osteoporosis (27–29). Use of glucocorticoid is associated with reduction in BMD (27), while use of tumor necrosis factor inhibitors is associated with increase in BMD in RA patients (28). Similarly, in our multivariable analysis, use of tumor necrosis factor inhibitor was associated with a higher increase of 1-year % change in lumbar spine BMD (β = 2.89 [95% CI: 0.93–4.85], *p* = 0.004), while the cumulative glucocorticoid dose between BMD tests was associated with a lower increase of 1-year % change in femoral neck BMD (β = −0.001 [95% CI: −0.002 to −0.001], *p* = 0.001) (Supplementary Table 1). In regard to csDMARDs, leflunomide may possibly be associated with an increase in lumbar spine BMD (29). In the present study, use of leflunomide had a positive effect on lumbar spine BMD (β = 1.02) as well, although it failed to reach statistical significance (95% CI: −1.01 to 3.04, *p* = 0.324) (Supplementary Table 1). We presume that this is because the outcome parameter was used as a continuous variable in the present study whereas in the previous study the outcome parameter was used as a categorical variable (29).

The present study had some limitations. First, this was a retrospective study, and data about the lifestyle habits of each patient, such as diet, alcohol intake, and exercise, were not available. The risk of confounding caused by these undetermined variables might exist. Further, due to the nonrandomized retrospective group allocation, possibility of selection bias cannot be excluded. Second, data about vitamin D levels were missing. Owing to the lack of these data, the proportion of patients with vitamin D deficiency at baseline was not identified and whether patients who are not deficient in vitamin D will also benefit from a higher dose of vitamin D supplementation was not evaluated. Third, although we found that ≥1,000 IU/day of vitamin D supplementation was associated with a higher increase in 1-year % change in BMD than 800 IU/day of supplementation, we were unable to determine the optimal dose of vitamin D supplementation because only a small fraction of patients received a dose of more than 1,000 IU/day. Further study investigating the optimal dose of supplementation of vitamin D, probably over 1,000 IU/day, is needed. Fourth, although we adjusted for the type of bisphosphonate in the multivariable analysis, bias which results from the higher use of risedronate in the 0-IU/day group and higher use of alendronate in the 400, 800, and ≥1,000-IU/day group cannot be fully adjusted.

In conclusion, we observed a significantly higher increase in lumbar spine and femoral neck BMD in 1 year in RA patients with osteoporosis receiving vitamin D supplementation compared with those who did not. The increase in BMD was the highest in patients who received a vitamin D supplementation dose of ≥1,000 IU/day. Our finding suggests that RA patients with osteoporosis who are receiving bisphosphonate may benefit more when a higher dose of vitamin D is supplemented than patients with osteoporosis in the general population.

## Data Availability Statement

All datasets presented in this study are included in the article/Supplementary Material.

## Ethics Statement

This study was approved by the Institutional Review Board (IRB) of Gangnam Severance Hospital (IRB No: 3-2020-0043) and Asan Medical Center in Seoul, South Korea (IRB No: 2018-0090). Requirement of informed consent was waived because of the retrospective nature of the study. Written informed consent for participation was not required for this study in accordance with the national legislation and the institutional requirements.

## Author Contributions

M-CP and Y-GK conceived the study. OK, M-CP, and Y-GK designed the study. OK, JO, M-CP, and Y-GK participated in the acquisition of data, data analyses, and data interpretation. OK, M-CP, and Y-GK wrote the manuscript. All authors contributed to the article and approved the submitted version.

## Conflict of Interest

The authors declare that the research was conducted in the absence of any commercial or financial relationships that could be construed as a potential conflict of interest.
