# Supplementary material for: Effect of Vitamin D Supplementation on Bone Mineral Density in Rheumatoid Arthritis Patients With Osteoporosis
Source: Front Med (Lausanne). 2020 Aug 21;7:443. doi: 10.3389/fmed.2020.00443 (PMC7473387; doi:10.3389/fmed.2020.00443)
Supplement: Supplementary file 1 [file Table_1.DOCX]

Supplementary Table 1. Coefficients of all variables included in the multivariable analysis

|  | Lumabar spine | | Femoral neck | |
| --- | --- | --- | --- | --- |
|  | β (95% CI) | P value | β (95% CI) | P value |
| Vitamin D supplementation |  |  |  |  |
| 0 IU/day | 0 (ref.) |  | 0 (ref.) |  |
| 400 IU/day | 2.51 (0.04–4.99) | 0.047 | 3.88 (1.83–5.94) | <0.001 |
| 800 IU/day | 2.90 (0.47–5.33) | 0.020 | 4.30 (2.25–6.35) | <0.001 |
| ≥1,000 IU/day | 6.01 (3.71–8.32) | <0.001 | 6.79 (4.87–8.71) | <0.001 |
| Age | -0.02 (-0.11−0.06) | 0.599 | -0.01 (-0.08−0.06) | 0.768 |
| Female | -0.97 (-3.77−1.83) | 0.497 | -1.73 (-4.11−0.66) | 0.154 |
| BMI | -0.03 (-0.25−0.19) | 0.782 | 0.12 (-0.06−0.30) | 0.204 |
| Total glucocorticoid dose prior to initial BMD test | 0.00 (0.00–0.00) | 0.397 | 0.00 (0.00–0.00) | 0.760 |
| Cumulative glucocorticoid dose between BMD tests | 0.000 (-0.001−0.001) | 0.643 | -0.001 (-0.002−-0.001) | 0.001 |
| Use of MTX | 0.84 (-1.42−3.10) | 0.464 | -0.40 (-2.27−1.46) | 0.670 |
| Use of HCQ | -0.57 (-2.25−1.11) | 0.503 | -0.89 (-2.28−0.50) | 0.208 |
| Use of SSZ | -0.41 (-2.73−1.91) | 0.726 | -0.06 (-1.97−1.86) | 0.955 |
| Use of LEF | 1.02 (-1.01−3.04) | 0.324 | 0.95 (-0.72−2.62) | 0.265 |
| Use of TAC | 0.70 (-2.23−3.63) | 0.637 | -1.11 (-3.53−1.31) | 0.365 |
| Use of TNFi | 2.89 (0.93−4.85) | 0.004 | 0.98 (-0.63−2.59) | 0.233 |
| Use of tocilizumab | 0.88 (-4.19−5.96) | 0.732 | -4.65 (-8.84−-0.46) | 0.030 |
| Use of abatacept | 1.28 (-2.10−4.66) | 0.456 | -0.98 (-3.77−1.81) | 0.489 |
| DAS28-ESR | -0.11 (-0.97−0.75) | 0.804 | 0.44 (-0.27−1.15) | 0.222 |
| Type of bisphosphonate |  |  |  |  |
| Risedronate | 0 (ref.) |  | 0 (ref.) |  |
| Alendronate | 1.48 (-0.75−3.73) | 0.191 | -0.50 (-2.38−1.38) | 0.600 |
| Ibandronate | -1.00 (-3.16−1.17) | 0.364 | 0.52 (-1.27−2.31) | 0.566 |

BMI, body mass index; BMD, bone mineral density; MTX, methotrexate; HCQ, hydroxychloroquine; SSZ, sulfasalazine; LEF, leflunomide; TAC, tacrolimus; TNFi, tumor necrosis factor inhibitor; DAS28-ESR, disease activity score 28-erythrocyte sedimentation rate
